# Extreme learning machine based optimal embedding location finder for image steganography

**DOI:** 10.1371/journal.pone.0170329

**Published:** 2017-02-14

**Authors:** Hayfaa Abdulzahra Atee, Robiah Ahmad, Norliza Mohd Noor, Abdul Monem S. Rahma, Yazan Aljeroudi

**Affiliations:** 1 Foundation of Technical Education, Higher Education and Scientific Research, Baghdad, Iraq; 2 Department of Engineering, UTM Razak School of Engineering and Advanced Technology, UTM Kuala Lumpur, Kuala Lumpur, Malaysia; 3 Computer Science Department, University of Technology, Baghdad, Iraq; 4 Department of Mechanical Engineering, International Islamic University of Malaysia, Kuala Lumpur, Malaysia; Jiangnan University, CHINA

## Abstract

In image steganography, determining the optimum location for embedding the secret message precisely with minimum distortion of the host medium remains a challenging issue. Yet, an effective approach for the selection of the best embedding location with least deformation is far from being achieved. To attain this goal, we propose a novel approach for image steganography with high-performance, where extreme learning machine (ELM) algorithm is modified to create a supervised mathematical model. This ELM is first trained on a part of an image or any host medium before being tested in the regression mode. This allowed us to choose the optimal location for embedding the message with best values of the predicted evaluation metrics. Contrast, homogeneity, and other texture features are used for training on a new metric. Furthermore, the developed ELM is exploited for counter over-fitting while training. The performance of the proposed steganography approach is evaluated by computing the correlation, structural similarity (SSIM) index, fusion matrices, and mean square error (MSE). The modified ELM is found to outperform the existing approaches in terms of imperceptibility. Excellent features of the experimental results demonstrate that the proposed steganographic approach is greatly proficient for preserving the visual information of an image. An improvement in the imperceptibility as much as 28% is achieved compared to the existing state of the art methods.

## Introduction

Over the decades, the ever-escalating advancements of communication technology allowed the free transferring and sharing of confidential information over the complex internet network. This free sharing of sensitive information in the form of data files, and video/audio recordings posed severe security threats. The preservation of users’ privacy is repeatedly threatened by the highly sophisticated and deceptive phishing attacks. Thus, absolute protection of sensitive data communication from unauthorized accesses or attacks is demanded.

Presently, the secured communication is achieved via mathematical models assisted cryptographic and steganographic techniques. Ironically, cryptography being the encryption of a plain-text for generating the cipher-text does not obscure the data existence. It rather makes the data incomprehensible to protect the secret message from attacks or unauthorized access. For absolutely secured information communication, the limitations of cryptography are surmounted by introducing a new technique called steganography. However, most of the conventional steganographic techniques suffer from high computational loads when selecting the best location for concealing the message in the host medium with minimal deformation. This shortcoming can be overcome by introducing the neural network (NN) based steganographic technique, where the NN uses a distributed representation to store the learning knowledge. Thus, accessing the concealed data without knowing the topology of the NN appears practically infeasible [1]. Although some researchers prefer models with interpretability power such as explicit mathematical or statistical models or even heuristically encoded models such as fuzzy models, it has been proved that black box type of models when learning is feasible have more capability of capturing complicated knowledge and proving functionality in real world type of systems [2][3][4]. Such black box models have dramatically proved high efficiency in the state of the art of speech recognition, visual object recognition and many other fields [5].

Using information-hiding protocols, the steganographic technique embeds the message into a cover medium to keep the hidden data from being detected. This cover medium may be an image, video, or audio file. Among various steganographic techniques image steganography (concealing data into an image) is most popular and widely used because it allows an easy exchange of vast amount of images via the internet [6]. On top, the image steganography assisted hidden data cannot be recognized through the visual inspection [7]. Lately, in the image steganography domain the heuristic searching optimization became attractive [5]. Despite much research achieving an efficient steganographic algorithm for finding the best embedding location with reduced computational time expenses remains challenging.

Depending on embedded locations, the image steganographic algorithms are categorized into spatial[8][9] and frequency domain embedding. The later one is also called transform-domain embedding [10]–[13]. In the spatial domain, the least significant bit (LSB) based steganography [8][9] is the most extensively used method [14], where the carrier or cover image LSB is applied to conceal the secret message. Conversely, in the least significant bit replacement (LSBR) based steganography, the hidden secret message can be uncovered by the existing steganalysis methods [15][16]. Thus, it is weak against visual and statistical attacks. The least significant bit matching (LSBM) method also called ± embedding method provides better security than LSBR. However, it is incompatible for most of the model-preserving steganographic techniques [17]. Despite their high capacity the spatial-domain techniques are not robust against image-processing operations, noise attacks, lossy compression, and filtering. Furthermore, they offset the statistical properties of the image due to the sole usage of the BMP format.

As aforementioned, in frequency-domain steganography the secret data are concealed in the significant parts of the cover image. This domain is comprised of several transforms such as discrete cosine transforms (DCT), discrete wavelet transforms (DWT), and discrete Fourier transforms (DFT). These transforms are used as media for hiding a message into an image [18]. Although both DWT and DCT have relatively smaller capacities but the former one is superior in terms of robustness against image-processing operations, statistical and noise attacks as well as distortion [19]. Thus, the steganographic techniques in the frequency-domain possess better immunity to attacks than the one spatial-domain. The limitations involving the spatial-domain techniques are overcome using frequency-domain. Numerous researches are performed with DWT [10], [12]. The presence of rounding error in the inverse DFT make it disadvantageous for steganographic applications [20]. Table 1 presents a brief summary of embedding the secret information in spatial or frequency domain.

**Table 1:** The embedding domain for the existing state of the art methods.

Author(s)	Domain and Technique	Pros	Cons
**Banerjee, Bhattacharyya, and Sanyal 2013**	Spatial–LSB	Capable of extracting the secret message without the cover image	Capacity issue has not addressed
**Pevny, Filler, and Bas 2010**	Spatial–LSB	Allows the embedder to conceal seven times longer message with same security	Applied theoretically and did not test by real data such as text or images
**Wu, Hsien C. et al. 2009**	Spatial–LSB	High payload in cover image	Unsatisfied image quality
**Luo, Huang, and Huang 2010**	Spatial-LSB	The visual quality and security have been improved significantly compared to conventional LSB	Did not tested against image processing or statistical analysis
**Islam and Gupta 2014**	Spatial–LSBM	Better security than LSBR	Conflicting for most of the model-preserving steganographic techniques
**Abdelwahab and Hassaan 2008**	Frequency–DWT	Does not require the original cover image to extract the embedded secret image.	Did not tested for text into image.
**Prabakaran and Bhavani 2012**	Frequency-DWT	Hiding a large-size secret image into a small-size cover image.	The quality of stego-image is not satisfied.

Some researchers have combined the spatial and frequency domains. The [21][22] introduced a framework for optimizing the adaptive distortion function to achieve minimal statistical detectability. The [23] improved the detection percentage and classified the images as stego or clean. Furthermore, spatial or frequency domain techniques are integrated with other techniques including artificial NN (ANN), genetic algorithm (GA), or both to attain enhanced steganographic performances. Spatial-domain based GAs are used [1], [24] to minimize the distortion and. GA and ANN are used [25] to accelerate the training speed. Frequency-domain ANN is used [26] to augment the embedding capacity. Spatial domain based ANN is utilized [27] to realize good approximation capacity, faster convergence, and a more stable performance surface. This type of ANN is also used [28] to increase the approximation capacity and minimize distortion.

The ANN is also used with steganography for message embedding [25], where the secret message is assumed to represent an image. This allowed the steganographer to change the message data freely provided the visual information is preserved. However, this assumption is not applied to the text messages. Meanwhile, ANN is also used for digital watermarking to authenticate the image [29], in which concealing a secret message is not required [30]. ANN is employed for the capacity maximization [28], steganographic content detection [31–33], identification of the embedded data in an image when applied to steganalysis or as a classifier and determination of the lower and upper bounds of embedding capacity [34]. Likewise, GAs are used in steganography for diverse purposes. GA is used to model the steganography problem [24] for search and optimization. Besides, for optimization with minimum distortion the GAs are utilized, where a stego image closer to the cover image is obtained [1], [35]. The [11] presents DCT with Markov as a detection and classifier for images. Table 2 summarizes different embedding techniques with combined spatial and frequency domains.

**Table 2:** The combined spatial and frequency domains with different embedding techniques for the existing state of the art methods.

Author(s)	Domain and Technique	Pros	Cons
**Tomás Filler and Fridrich 2011**	Frequency-DCT and Spatial	Strong against many types of steganalysis	High complexity
**Tom Filler, Judas, and Fridrich 2011**	Frequency-DCT and spatial	The methods are not limited to binary embedding and allow the embedder to choose the amplitude of embedding changes dynamically based on the cover-image content.	Focus on payload aspects rather than embedding
**Pathak and Selvakumar, 2014**	Frequency-DCT and Spatial	It is used as a classifier and embedding.	This method omitted some features of images.
**Iranpour and Rahmati 2014**	Spatial and GA	Enhancing the security by minimize the distortion.	Omitted the optimum number of blocks as well as their sizes.
**El-Emam and AL-Zubidy 2013**	Frequency GA and ANN	Allowed the steganographer to change the message data freely provided the visual information is preserved.	Omits the text steganography.
**Tsai et al. 2009**	Frequency and ANN	Augment the embedding capacity and supports true-color secret image with size constraint on shares.	Hiding small image into large image.
**Husien and Badi 2014**	Spatial and ANN	Good approximation capacity, faster convergence, and more stable performance surface.	Did not present numerical comparisons with other works.
**Ghaleb Al-jbara, Mat Kiah, and Jalab 2012**	Spatial-LSB and ANN	Increases the approximation capacity.	PSNR and MSE are not satisfied and did not tested against image processing.
**El-Alfy 2013**	Spatial domain-PVD and ANN	99% rates of detection have been achieved.	Applied only in transformed domain.
**Pratt, Konda, and Chu 2008**	Spatial-LSB, and ANN	It is especially challenging when the embedding rate is low, such as below 10 percent of all embedded data.	It is used as a steganalysis and not as embedding. Some error rates have been addressed in extracting the embedded data.
**Nazeri and Kanan 2014**	Spatial domain and GA	It is modeling the steganography problem as a search and optimization problem.	Did not tested against image processing or any statistical analysis attack.
**Roy and Laha 2015**	Spatial- LSB and GA	High security and robustness.	The image quality (PSNR) is not satisfied.
**Cho, Seongho, Byung-Ho Cha, Martin Gawecki, and C.-C. Jay Kuo 2013**	Frequency–DCT and Markov	Tested in terms of spatial and frequency domains	Using as a classifier not as embedding

Lately, the learning ability of NNs is exploited to expand the optimization potential of conventional data-hiding techniques. In steganography, ANN is used either for the classification of the stego image or for the detection of the embedded data in an image. We intend to reduce the distortion in a stego image as much as possible by appropriately selecting the location in the image for messages embedment. Theoretically, an ELM demonstrates a good generalization performance and universal approximation at extremely fast learning speeds. It can be used for either classification or regression purposes [36]. Inspired by such notable advantages, we propose an ELM-based supervised mathematical model called Optimal Embedding Location Finder (OELF) for image steganography. In addition, a novel fusion metric (*fusion1*) is introduced for the training in the regression mode to realize the best performance metric for steganography. Another novel fusion metric (*fusion2*) is developed for evaluating the results. To the best of our knowledge, for the first time we use the machine learning to determine the best location with least sensitive area for embedding.

This paper is organized as follows. Section 2 depicts the proposed OELF model. Section 3 highlights the detail mathematical background of steganography. Section 4 describes the proposed methodology. Section 5 explains the experimental results with various attributes. Section 6 concludes the paper with further outlook.

## Optimal Embedding Location Finder (OELF) model

Most traditional steganographic methods embed the message into an image by ignoring the significance of the image’s spatial features. Nevertheless, the identification of best embedding location is critically decided by the message homogeneity and other texture features [37] of the blocks. A location having least image distortion is considered to be the optimum one. To protect the embedding process from a steganalysis, any form of distortion in the image must be minimized after the payload is inserted. Furthermore, the cover image and stego image must be approximately identical both visually and statistically. The selected area and the embedding method are the primary factors that affect the distortion. Based on OELF model an ELM is proposed for finding the best embedding location. It is worth noting that ELM is beneficial due to its universal approximation capacity which allows rapid training with good over-fitting avoidance than other classical NN based approaches [36]. Thus, a modified ELM is used to train a single-hidden-layer NN with a varying number of neurons. Appendix A provides a short depiction of ELM.

## Background of steganography modeling

As mentioned earlier, OELF locates the most suitable window for embedding the secret message into the image without affecting its visual features. Initially, the image is partitioned into (8 × 8) block pixels and one bit of the message is inserted into each block. Depending on the message size, the image is then partitioned into overlapping square windows to embed the message. The features of contrast (*C*), energy (*Enr*), homogeneity (*H*), entropy (*Ent*), correlation (*Corr*), standard deviation (*Std*), and the mean (*M*) of each square window are calculated using:

$$C=\sum_{i,j}{\left|i-j\right|}^{2}\;p\left(i,j\right)$$ (1)

$$Enr=\;\sum_{i,j}p{\left(i,j\right)}^{2}$$ (2)

$$H=\sum_{i,j}\frac{1}{1-{\left(i-j\right)}^{2}}p\left(i,j\right)$$ (3)

$$Ent=\;-\sum_{i,j}p\left(i,j\right)\text{log}(\left(i,j\right)$$ (4)

$$Corr=\frac{cov\left(Cover\_image,\;Stego\_image\;\right)}{\left\|Cover\_image\right\|\;\;\left\|Stego\_image\right\|}$$ (5)

where *i* and *j* are the horizontal and vertical pixel coordinates, respectively, and *p* is the pixel value.

$$cov\left(x,y\right)=\frac{1}{N}\sum_{i=1}^{N}\left(x_{i}-E\left(x\right)\right)\left(y_{i}-E\left(y\right)\right)$$ (6)

where *N* is the number of the window pixels.

$$Std=\sigma_{xy}=\sqrt{cov\left(x,y\right)}$$ (7)

$$M=E\left(x\right)=\frac{1}{N}\sum_{i=1}^{N}x_{i};\;E\left(y\right)=\frac{1}{N}\sum_{i=1}^{N}y_{i}$$ (8)

After calculating the window features and embedding the message in the corresponding window, the resultant imperceptibility is represented using one of three metrics including correlation, MSE, and SSIM. The expression for MSE and SSIM yields:

$$MSE=\frac{1}{N\times M}\sum_{i=0}^{N-1}\sum_{j=0}^{M-1}{\left[Cover\_image\left(i,j\right)-Stego\_image\left(i,j\right)\right]}^{2}$$ (9)

where *N* and *M* are the length and width of the image, respectively.

$$SSIM=\frac{\left(2\mu_{x}\mu_{y}+C_{1}\right)\left(\;\sigma_{xy}^{2}+C_{2}\right)}{\left(\mu_{x}^{2}+\mu_{y}^{2}+C_{1}\right)\left(\sigma_{x}^{2}+\sigma_{y}^{2}+C_{2}\right)}$$ (10)

where μ_*x*_ and μ_*y*_ are the local mean, σ_*x*_ and σ_*y*_ are the standard deviation, σ_*xy*_ is the cross-covariance, *C*_1_ and *C*_2_ are constants.

## Methodology

The following subsections describe the detailed methodology including the input (host or cover image), the message to be embedded in the image, the output (stego image) and the evaluation metrics of imperceptibility.

### Input and output determination

**Fig 1:**
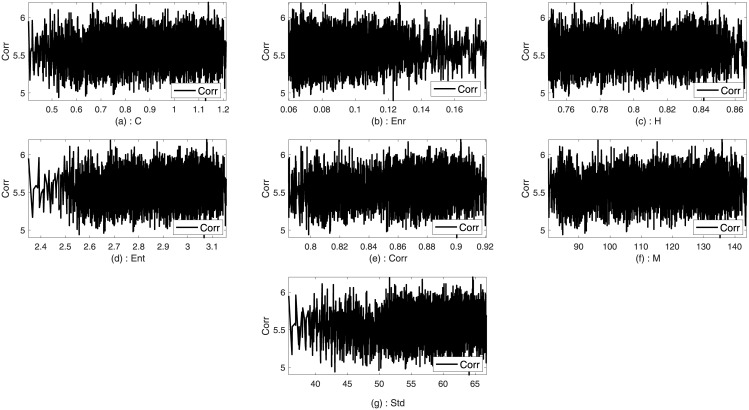
Relationship of the correlation metric to the texture features (a) contrast, (b) energy, (c) homogeneity, (d) entropy, (e) correlation, (f) mean, and (g) standard deviation for Lena image.

**Fig 2:**
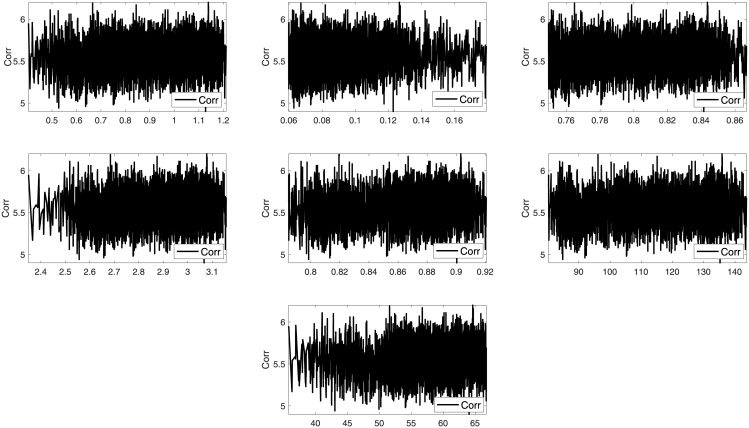
Relationship of the MSE metric to the texture features (a) contrast, (b) energy, (c) homogeneity, (d) entropy, (e) correlation, (f) mean, and (g) standard deviation for Lena image.

**Fig 3:**
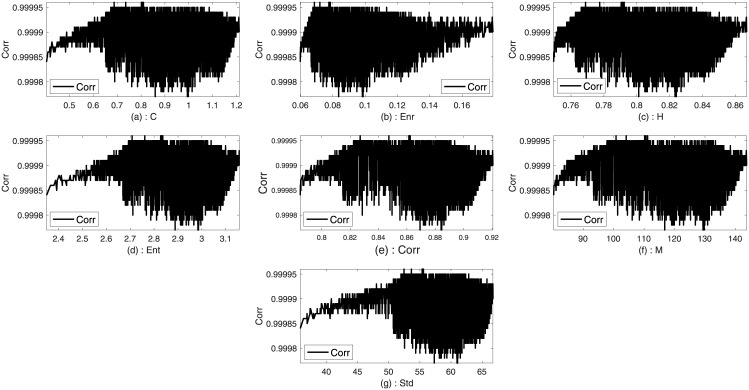
Relationship of the SSIM metric to the texture features (a) contrast, (b) energy, (c) homogeneity, (d) entropy, (e) correlation, (f) mean, and (g) standard deviation for Lena image.

**Fig 4:**
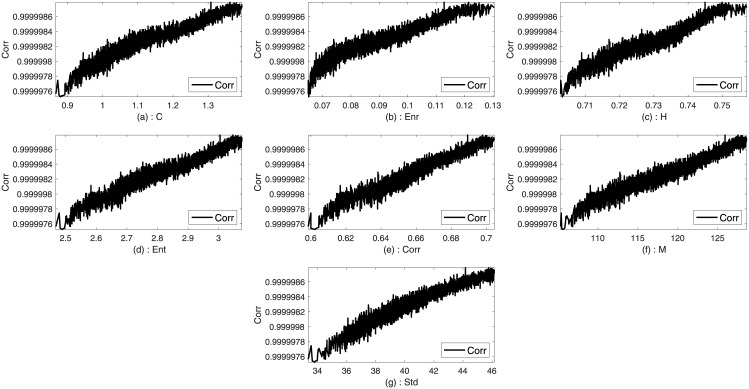
Relationship of the correlation metric to the texture features (a) contrast, (b): energy, (c) homogeneity, (d) entropy, (e) correlation, (f) mean, and (g) standard deviation for Sails image.

**Fig 5:**
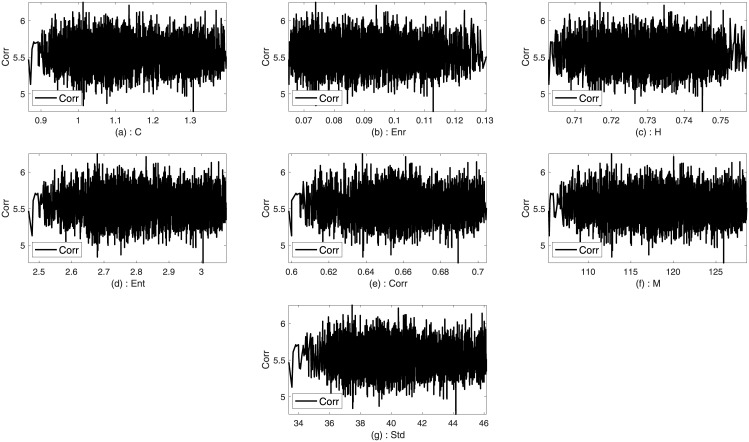
Relationship of the MSE metric to the texture features (a) contrast, (b) energy, (c) homogeneity, (d) entropy, (e) correlation, (f) mean, and (g) standard deviation for Sails image.

**Fig 6:**
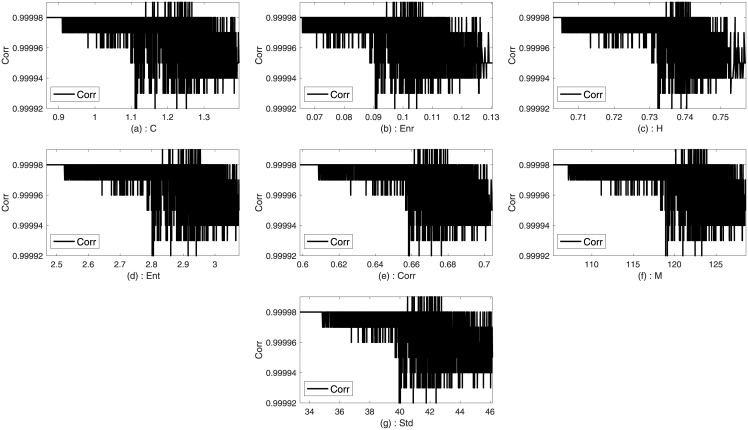
Relationship of the SSIM metric to the texture features (a) contrast, (b) energy, (c) homogeneity, (d) entropy, (e) correlation, (f) mean, and (g) standard deviation for Sails image.

Two images such as Lena and Sails from the standard database are used to analyze the trends between the imperceptibility and the texture features of the image. Imperceptibility is measured in terms of correlation, MSE, and SSIM between two corresponding square windows for the host and stego images with respect to the extracted features. Figs 1–6 show the trends of the imperceptibility of the Lena and Sails images after the message is embedded into a square window regarding the corresponding texture features in this window. It is evident that all the features (contrast, energy, homogeneity, entropy, correlation, entropy, and Std) are strongly correlated. The occurrence of less variability in the imperceptibility correlation with respect to the set of features implies their equivalent usage in the machine learning model.

Table 3 summarizes the trends of imperceptibility to texture feature.

**Table 3:** Trends of the imperceptibility to the texture feature for the Lena and Sails images.

Features	Measures		
	Correlation	MSE	SSIM
**Contrast**	Positive	No trend	Negative
**Energy**	Positive	No trend	Negative
**Homogeneity**	Positive	No trend	Negative
**Correlation**	Positive	No trend	Negative
**Mean**	Positive	No trend	Negative
**Standard deviation**	Positive	No trend	Negative
**Entropy**	Positive	No trend	Negative

A detail analysis of such trends between the imperceptibility and the texture features of the image allowed us to determine the possible causality among them. Thus, the machine learning is designed with an optimized embedder or steganographer.

### Model design

The following steps are adopted to develop the proposed model:

1. Partitioning of the (*N* × *M*) host image into (*K* × *L*) pixel non-overlapping sub-blocks, where (*K* = *L* = 8).
2. Determination of the number of blocks needed to embed the message according to the message bits’ size *m*.
3. Determination of the minimum square window size (*SWS*) from the image that contains the required blocks. The SWS is calculated using:
  $$\begin{array}{cc} SWS=8\left\lceil \sqrt{m}\right\rceil \;\;\times \;\;8\left\lceil \sqrt{m}\right\rceil & \text{Pixels} \end{array}$$ (11)
4. Creation of raw data set of the square windows with a scanning resolution of 4 pixels NOS. The size of the data set is:
  $$NOS=\left(\left(\frac{N-SWS}{4}\right)+1\right)\times \left(\left(\frac{M-SWS}{4}\right)+1\;\right)$$ (12)

where *N* and *M* are the length and width of the image, respectively, and SWS is the square window size.

### Data set preparation

**Fig 7:**
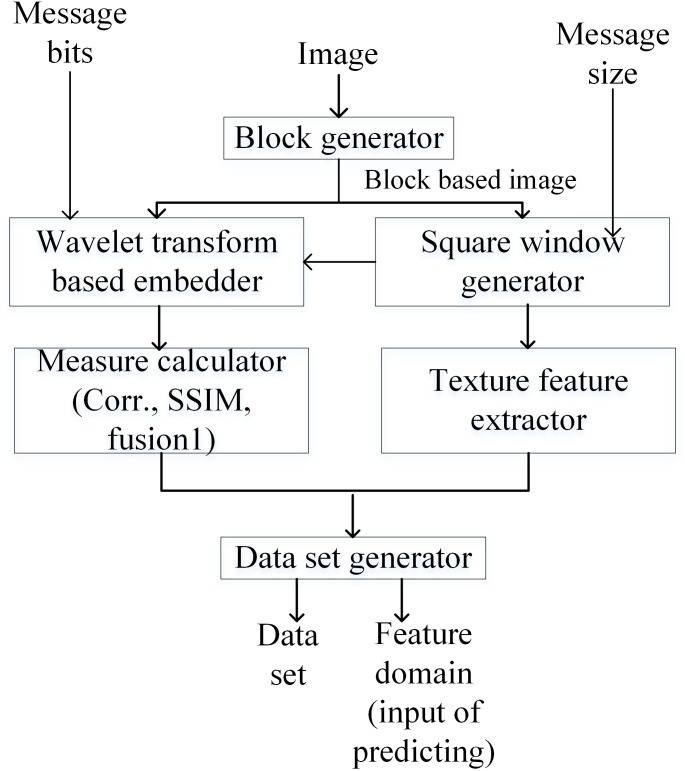
Construction of data set and feature domain.

Fig 7 illustrates the schematic framework for the creation of the learning data set and the feature domain prior to the ELM training and testing. The texture feature extraction, metric calculation and embedding are performed for building the learning data set. It is customary to explain briefly the embedding and the feature extraction procedure.

#### Wavelet transform based embedding

As aforementioned, the message must be embedded into its corresponding square window for each square window in the data set. The learning data are extracted from the raw data set using the embedding process and the calculation of the resultant visual imperceptibility metrics. To achieve this goal, the following steps are executed:

1. For message bit one, the value is quantized to the nearest even number with the index (8, 8) in the corresponding block. Otherwise, it is quantized to the nearest odd number.
2. The wavelet transform for each sub-block is computed by inverting the wavelet.
3. The wavelet is transformed to its corresponding spatial domain.
4. The embedding process is repeated until the final bit of the message is embedded.
5. For each square window, the corresponding visual metrics are calculated. These metrics include correlation, MSE, SSIM, and fusion1. The expression for *fusion*1 yields:

fusion1=correlation × SSIM  (13)

#### Texture feature extraction

The texture features are extracted using the following steps:

1. The co-occurrence matrix is built for each square window whose sub-blocks are used for embedding the message bits.
2. The feature function (contrast, energy, homogeneity, entropy, correlation, and standard deviation) of the co-occurrence matrix is computed for each square window. The expression for *features* yields

features=(C, Enr, H, Ent, Corr, M, Std) (14)

### Extreme Learning Machine (ELM)

#### ELM training

The prepared data is represented the matrix form

$$X=\left(f_{1j},\;f_{2j},\ldots ,f_{7j};\;y_{ij}\right),\;\text{with}\;j=1,\ldots ,n$$

where *n* is the number of square windows, *f*_1*j*_, *f*_2*j*_,…, *f*_7*j*_ are the extracted features, *y*_*ij*_ is the corresponding output metrics, and *i* = 1, 2, 3, 4 correspond to the Corr, MSE, SSIM, and fusion1, respectively.

A neural network of *ñ* hidden neurons is built and trained on a part of *X* to predict *y*_*i*_. Furthermore, the training and the testing phases are validated using the RMSE before applying the ELM-based model. Now we turn our attention in determining the optimal training percentage and the optimum number of neuron.

#### RMSE for training and testing

The OELF being a supervised model the authentication of the training and testing phases are necessary. They play a decisive role in the proposed model. In the present case, OELF is trained to predict the visual imperceptibility metrics (Corr and SSIM) and the fusion1 metric. The RSMEs of the proposed OELF model for the training and testing phase are computed to evaluate its predictability performance. Table 4 summarizes the RSME values of the square window for each of the similarity metrics. The computed RSMEs for all the metrics in both the training phase and testing phase with different images are discerned to be approximately zero, indicating the suitability of the proposed model.

**Table 4:** RMSEs for the training phase and testing phase for different images.

Images	Measure	RMSE (Training phase)	RMSE (Testing phase)
**Lena**	Corr	0.0000002592	0.0000002604
	MSE	0.000183980	0.0001953800
	SSIM	0.0000060068	0.0000063730
	Fusion1	0.0000059813	0.0000063790
**Sails**	Corr	0.0000013995	0.0000014011
	MSE	0.000179340	0.0001922900
	SSIM	0.0000097757	0.0000088329
	Fusion1	0.0010000000	0.0011000000
**Baboon**	Corr	0.0000010623	0.0000010641
	MSE	0.000193010	0.0002089800
	SSIM	0.0000041315	0.0000042289
	Fusion1	0.0000022554	0.0000023760
**4.2.01**	Corr	0.0000000833	0.0000000874
	MSE	0.000193390	0.0002117400
	SSIM	0.0000891080	0.0000968680
	Fusion1	0.0000891170	0.0000968790

#### Developed ELM training

A number of issues need to be addressed when using ELM. First, an appropriate training–testing ratio has to be determined accurately to avoid over-fitting for using a high training percentage and under-fitting for using a low training percentage. Second, the ELM does not provide the user with the exact number of neurons to be selected for building the network structure. Moreover, the performance of the model depends on the accurate determination neurons number, where a large (small) number of neurons lead to over (under) fitting [38] [2].

**Fig 8:**
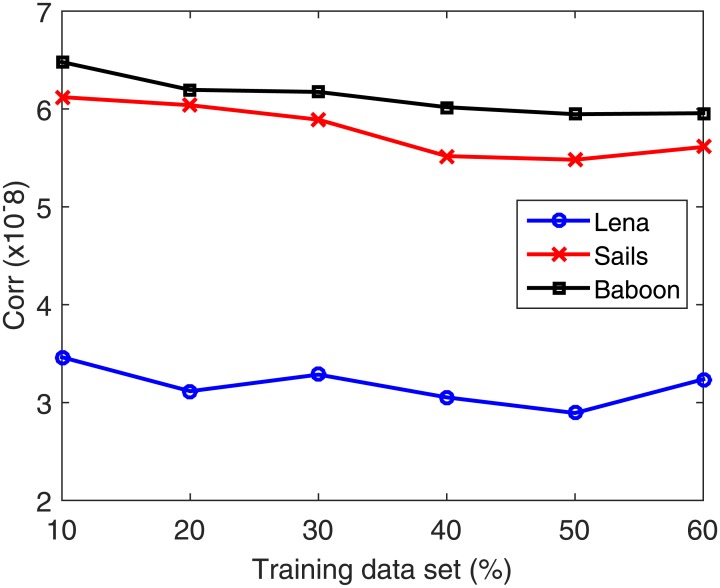
Training data set percentage dependent variation of Corr for the Lena, Sails, and Baboon images.

**Fig 9:**
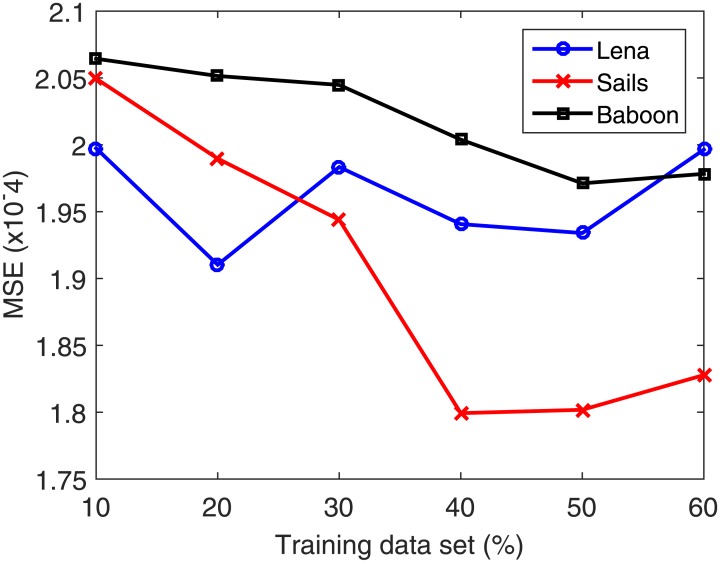
Training data set percentage dependent variation of MSE for the Lena, Sails, and Baboon images.

**Fig 10:**
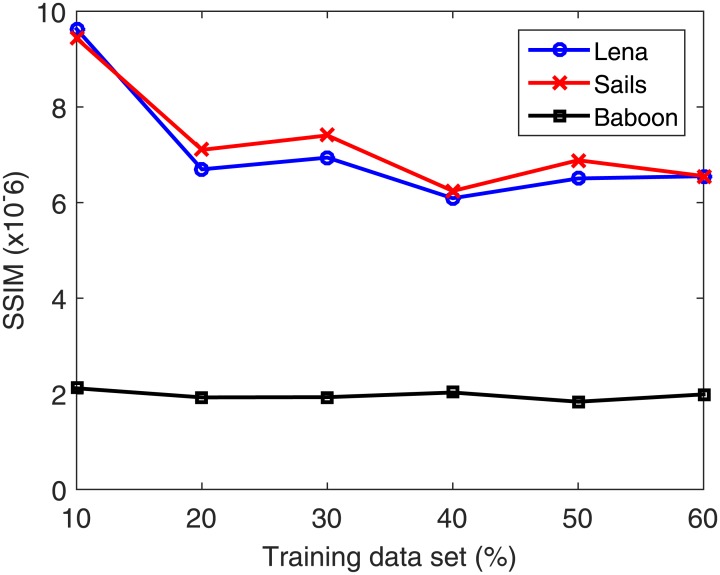
Training data set percentage dependent variation of SSIM for the Lena, Sails, and Baboon images.

**Fig 11:**
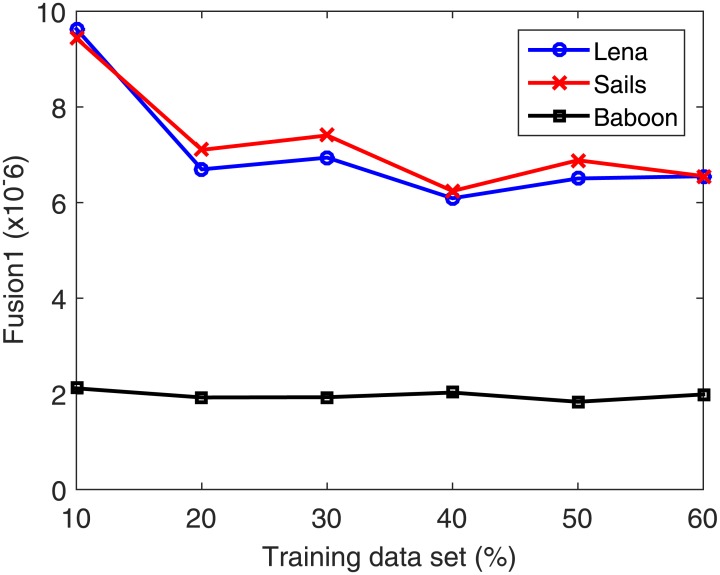
Training data set percentage dependent variation of fusion1 for the Lena, Sails, and Baboon images.

The used data set is partitioned into 50% training and 50% testing. Next, the number of neurons is increased from 50 to 200 at a step of 5. In each case, the data set is partitioned into 80% for training and 20% for validation. Validation is performed on a part of the training data set because in the normal functioning mode of the system the testing data set is unavailable. The number of neurons in the hidden layer corresponding to the best validation accuracy is then selected. Once the optimal number of neurons is selected, the search for the best training–testing ratio is performed by assigning a fixed testing data set size. Allocation of fixed percentage of the data for testing is required to avoid the bias in the RSME with increasing testing data set. Afterward, the percentage of the training data is increased from 10% to 60% for validating each case using the validation part composed of 20% of the training data set. From the total data set, 50% is found to be best for training. Table 5 summarizes the training data set (%) dependent accuracy levels for the Lena, Sails and Baboon images. Figs 8–11 displays the training data set percentages dependent variation in the Corr, MSE, SSIM, and fusion1 values between the host and stego images (Lena, Sails, and Baboon).

**Table 5:** Accuracy levels of the different training data set percentages for the Lena, Sails and Baboon images.

Images	Training (%)	Sample No.	Corr	MSE	SSIM	fusion1
**Lena**	10	504	0.000000034633	0.00019974	0.0000096058	0.0000096302
	20	1008	0.000000031163	0.00019103	0.0000066814	0.0000066951
	30	1512	0.000000032858	0.00019834	0.0000069203	0.0000069401
	40	2016	0.000000030532	0.00019407	0.0000066675	0.0000066905
	50	2520	0.000000028931	0.00019340	0.0000064897	0.0000065042
	60	3025	0.000000032376	0.00019964	0.0000064997	0.0000065511
**Sails**	10	504	0.000000061209	0.00020495	0.0000107230	0.0000094358
	20	1008	0.000000060383	0.00019897	0.0000073274	0.0000071024
	30	1512	0.000000058905	0.00019439	0.0000074816	0.0000074046
	40	2016	0.000000055183	0.00017993	0.0000062795	0.0000062419
	50	2520	0.000000054812	0.00018018	0.0000067782	0.0000068851
	60	3025	0.000000056117	0.00018278	0.0000064654	0.0000065537
**Baboon**	10	504	0.000000064792	0.00020644	0.0000026758	0.0000021155
	20	1008	0.000000061951	0.00020515	0.0000019249	0.0000019248
	30	1512	0.000000061739	0.00020448	0.0000018580	0.0000019298
	40	2016	0.000000060180	0.00020039	0.0000020036	0.0000020270
	50	2520	0.000000059464	0.00019712	0.0000018142	0.0000018343
	60	3025	0.000000059567	0.00019783	0.0000019505	0.0000019876

### Design and optimization of ELM

**Fig 12:**
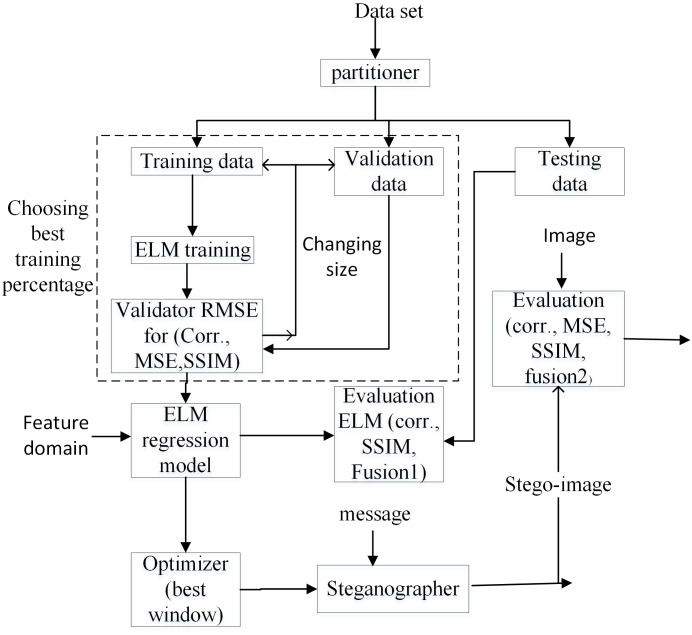
General framework of the proposed OELF model.

Fig 12 depicts schematically the framework of the proposed OELF model, which is achieved using the following steps:

1. The data set is partitioned into 50% for training as well as validation and 50% for testing.
2. The ELM regression model is designed based on the training data set (Appendix A) which is partitioned into 80% for training and 20% for validation.
3. The ELM regression model is further used to predict the best square window in terms of the fusion2 metric.
4. The embedding process is performed to insert the secret message into the identified optimum square window for generating the stego image.

Using the ELM training the message is embedded into each square window and all visual imperceptibility metrics are determined via fusion2 metric given by:

$$fusion2=\frac{\text{Corr }\times \text{SSIM}}{\text{MSE}}$$ (15)

## Experiments and results

**Fig 13:**
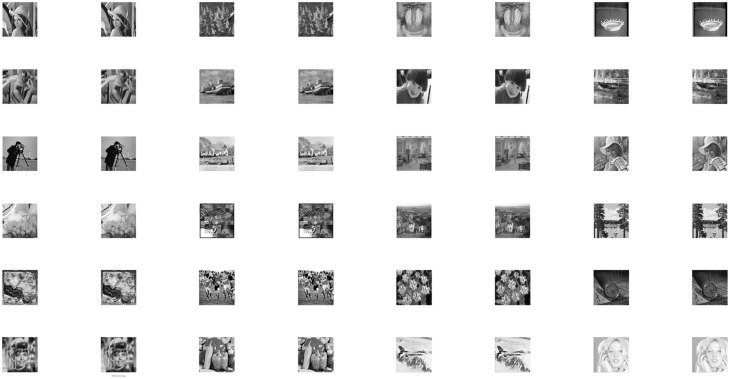
Achieved host (left) and stego (right) images.

Experiments are conducted on Intel^®^Core^™^ i7-2670QM CPU @ 2.20 GHz 6 GB RAM computer with 64-bit operating system. The proposed OELF model is evaluated using 24 gray scale images of size (512 × 512) pixels. Total 5041 square windows are obtained, in which square windows of (232 × 232) are used. The message of size 100 bytes is utilized for embedment. Fig 13 illustrates the tested images before (left panel of each image) and after (right panel of each image) embedding.

Table 6 enlists the RMSEs of the ELM prediction for the visual imperceptibility metrics of the host and stego images for 50% training data set.

**Table 6:** RMSE values obtained using the ELM model for various images.

Image	Corr	MSE	SSIM	fusion2
**Lena**	0.000000028931	0.00019340	0.0000064897	6.2242
**Sails**	0.000000054812	0.00018018	0.0000067782	5.9077
**Baboon**	0.000000059464	0.00019712	0.0000018142	6.3427
**4.2.01**	0.000000072910	0.00021492	0.0000897420	6.7471
**Barbara**	0.000000046185	0.00020671	0.0000038981	6.5776
**Boat**	0.000000046384	0.00018874	0.0000014097	6.1217
**Boy**	0.000000036993	0.00018259	0.0002314300	5.8925
**Bridge**	0.000000037995	0.00018965	0.0000006518	6.0393
**Cameraman**	0.000000034447	0.00019519	0.0000087432	6.3871
**Car**	0.000000050114	0.00019526	0.0003784400	6.1066
**Couple**	0.000000074732	0.00017851	0.0000070962	5.7333
**Elaine**	0.000000048013	0.00018282	0.0000011586	5.9634
**Fruits**	0.000000091042	0.00018445	0.0000388410	5.9403
**Fry mire**	0.000000029372	0.00020057	0.0010000000	6.0890
**Gold hill**	0.000000068636	0.00020280	0.0000048690	6.4077
**Lake**	0.000000028259	0.00019234	0.0000295420	6.0766
**Serrano**	0.000000027705	0.00021271	0.0012000000	6.1314
**Sport team**	0.000000018767	0.00019272	0.0018000000	5.9935
**Tulips**	0.000000041771	0.00020646	0.0000247010	6.7391
**Watch**	0.000000170190	0.00021892	0.0012000000	6.0348
**Zelda**	0.000000076098	0.00019957	0.0000061628	6.4019
**Pepper**	0.000000042533	0.00019457	0.0000098827	6.2634
**F16**	0.000000050038	0.00019940	0.0001149300	6.3402
**Tiffany**	0.000000138370	0.00018762	0.0001004100	5.9857

**Fig 14:**
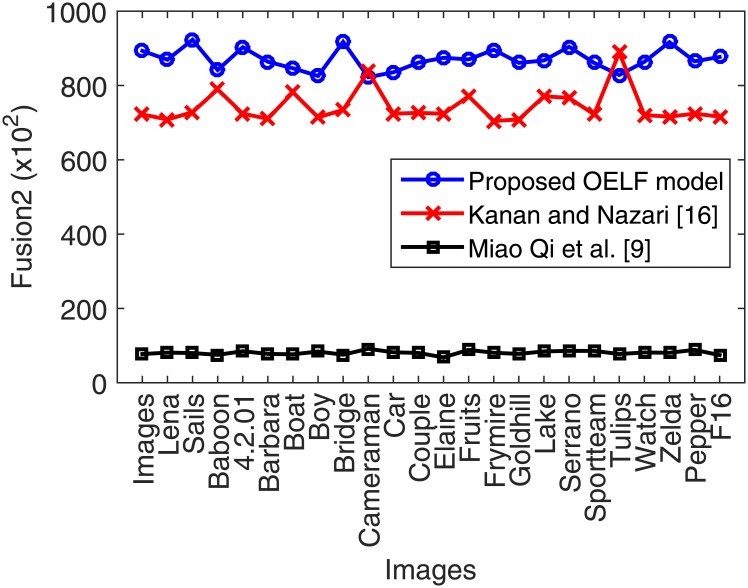
Performance of the proposed imperceptibility metric (*fusion2*).

The experimental results obtained using the proposed OELF model are compared (Table 7 and Fig 14) with the art-of-the existing methods [24], [39] in terms of the *fusion2* metric. OELF approach is found to outperform the other methods [24], [39] in terms of imperceptivity and *fusion2* measure which are nearly 28% and 114%, respectively. Thus, OELF is demonstrated to be a useful steganography technique for embedding text in images with minimum level of distortion. Furthermore, it requires only a small training part of the host image features.

**Table 7:** Comparison of the OELF model results with other existing models.

	Proposed OELF model				Kanan and Nazeri [24]				Miao Qi et al. [39]			
Image	MSE	Corr.	SSIM	Fusion2	MSE	Corr.	SSIM	Fusion2	MSE	Corr.	SSIM	Fusion2
Lena	0.001133	0.999999	0.999989	881.8879	0.001384	0.999999	0.999996	722.1569	0.012939	0.999998	0.999910	77.2759
Sails	0.001130	0.999999	0.999996	884.8709	0.001388	0.999999	0.999994	720.1871	0.012329	0.999995	0.999957	81.1051
Baboon	0.001126	0.999999	0.999997	887.8688	0.001372	0.999999	0.999998	728.1764	0.012512	0.999996	0.999994	79.9213
4.2.01	0.001199	0.999999	0.999948	833.4831	0.001266	0.999999	0.999981	789.5758	0.013305	0.999997	0.999937	75.1510
Barbara	0.001205	0.999999	0.999994	829.5649	0.001380	0.999999	0.999995	724.1509	0.011779	0.999997	0.999972	84.8887
Boat	0.001109	0.999999	0.999996	901.4161	0.001407	0.999999	0.999995	710.4141	0.012878	0.999997	0.999929	77.6436
Boy	0.001181	0.999999	0.999971	846.2843	0.001277	0.999999	0.999992	782.5134	0.012756	0.999998	0.999992	78.3917
Bridge	0.001064	0.999999	0.999998	939.5825	0.001399	0.999999	0.999994	714.2845	0.011908	0.999998	0.999988	84.0194
Camera-man	0.001099	0.999999	0.999995	909.6258	0.001361	0.999999	0.999994	734.2930	0.013244	0.999998	0.999928	75.4968
Car	0.001080	0.999999	0.999961	925.5033	0.001194	0.999999	0.999982	837.5063	0.010925	0.999997	0.999973	91.5281
Couple	0.001117	0.999999	0.999995	894.6849	0.001380	0.999999	0.999995	724.1515	0.012207	0.999996	0.999996	81.9194
Elaine	0.001239	0.999999	0.999995	806.5932	0.001377	0.999999	0.999995	726.1570	0.012451	0.999997	0.999947	80.3092
Fruits	0.001182	0.999999	0.999966	845.5976	0.001380	0.999999	0.999987	724.1452	0.014648	0.999997	0.999955	68.2634
Fry-mire	0.001273	0.999999	0.999600	784.9895	0.001296	0.999999	0.999995	771.0079	0.010742	0.999999	0.999995	93.0905
Gold- hill	0.001148	0.999999	0.999995	870.9060	0.001419	0.999999	0.999992	704.6828	0.012390	0.999997	0.999937	80.7041
Lake	0.001176	0.999999	0.999990	860.3891	0.001411	0.999999	0.999990	708.4908	0.012878	0.999998	0.999990	77.6484
Serrano	0.001162	0.999999	0.999817	860.3891	0.001296	0.999999	0.999993	771.0064	0.012512	0.999998	0.999994	79.9214
Sport -team	0.001155	0.999999	0.999542	864.7656	0.001304	0.999999	0.999993	766.4982	0.012023	0.999998	0.999976	83.1654
Tulips	0.001062	0.999999	0.999992	941.2638	0.001384	0.999999	0.999991	722.1538	0.011413	0.999998	0.999968	87.6121
Watch	0.001139	0.999999	0.999730	877.2379	0.001135	0.999999	0.999987	888.6120	0.012390	0.999996	0.999991	80.7084
Zelda	0.001165	0.999999	0.999986	858.0696	0.001388	0.999999	0.999983	720.1634	0.012268	0.999996	0.999910	81.5048
Pepper	0.001156	0.999999	0.999985	864.4361	0.001396	0.999999	0.999990	716.2335	0.012390	0.999997	0.999981	80.7077
F16	0.001115	0.999999	0.999976	896.1971	0.001380	0.999999	0.999990	724.1478	0.011291	0.999997	0.999960	88.5585
Tiffany	0.001131	0.999999	0.999960	884.0927	0.001396	0.999999	0.999985	716.2293	0.134440	0.999996	0.999991	74.3775

## Conclusion

Based on ELM, we proposed a novel OELF model to achieve high-performance image steganography. In this approach, a modified ELM algorithm is used to establish the supervised mathematical model for determining the optimum embedding image location with minimal distortion. The ELM is trained on an image part (or any host medium) and tested in the regression mode to select the best location for embedding the message. This allowed in achieving the best values of the predicted evaluation metrics. The training is performed based on a set of the extracted texture, statistical features, and their corresponding visual imperceptibility metrics using a part of the image. The trained model is further used for the performance optimization. The proposed model is demonstrated to outperform the existing state-of-the-art models. The excellent features of the results suggest that the present model may constitute a basis for the development of secured image steganography algorithm. It is worth to look at the robustness of the proposed method against various statistical attacks by incorporating a wider range of features. Also, it is good to further develop the model to have more degree of freedom in terms of the region finding by defining the region analytically instead of explicit geometrical definition (block region). Other worthy development is to create an index for ranking the solution based on Pareto efficiency.

## Appendix A

For ELM training, the used data is combined with *n* arbitrary distinct square windows (x_j_, t_j_) with j = 1,…*n*, *x*_*j*_ = (*x*_*j*1_, x_*ij*_,…, *x*_*jn*_) denotes the input vector and t_j_ denotes the target. It is possible to model the standard Single Hidden Layer Feed Forward Network (SLFN) with an activation function g(x) and *ñ* hidden layer neurons via:

$$\sum_{\text{i}=1}^{Ñ}\beta_{\text{i}}\text{g}\left({\text{w}}_{\text{i}}{\text{x}}_{\text{j}}+{\text{b}}_{\text{i}}\right)={\text{t}}_{\text{i}}$$ (A.1)

where j = 1,…*n*, *w*_*i*_ = (*a*_*i*1_, *a*_*i*2_,…, *a*_*in*_)^*T*^, b_i_ is the threshold (biases) of the i^th^ hidden node, and β_i_ is the weight connecting the i^th^ hidden node and the output.

The above equation is compact form yields:

Иβ=T (A.2)

where И = И(a_1_, a_2_,…, a_*ñ*_, x_1_, x_2_,…, x_N_, b_1_, b_2_,…b_*ñ*_)

$$И=\left[\begin{array}{ccc} \text{g}\left({\text{a}}_{1}{\text{x}}_{1}+{\text{b}}_{1}\right) & \cdots & \text{g}\left({\text{a}}_{\tilde{n}}{\text{x}}_{1}+{\text{b}}_{\tilde{n}}\right)\\ \vdots & \ddots & \vdots \\ \text{g}\left({\text{a}}_{1}{\text{x}}_{n}+{\text{b}}_{1}\right) & \cdots & \text{g}\left({\text{a}}_{\tilde{n}}{\text{x}}_{n}+{\text{b}}_{\tilde{n}}\right) \end{array}\right]$$ (A.3)

with

$$\begin{array}{cc} \beta =\left(\begin{array}{c} \beta_{1}\\ \vdots \\ \beta_{ñ} \end{array}\right) & \text{T}=\left(\begin{array}{c} {\text{t}}_{1}\\ \vdots \\ {\text{t}}_{n} \end{array}\right) \end{array}$$ (A.4)

where И is called the hidden layer output matrix of the neural network and T is the target vector.

One can prove that if the activation function is differentiable then the required number of the hidden layer neurons is lower than the data size or *ñ* < *n*. The training of the neural network is achieved via the following steps:

1. Assigning random weights (*w*_*i*_) and biases (b_i_).
2. Calculating the hidden layer output matrix.
3. Computing the output weights (β) via:

$$\beta ={И}^{\text{T}}\text{T}$$ (A.5)

where И^T^ is the Moore-Penrose generalized inverse of hidden layer output matrix.

## Supporting information

S1 File Original data for Figs 1–6,8–11 and 14. (XLSX)
